# L-Carnitine Reduces Oxidative Stress and Promotes Cells Differentiation and Bone Matrix Proteins Expression in Human Osteoblast-Like Cells

**DOI:** 10.1155/2019/5678548

**Published:** 2019-01-20

**Authors:** Ileana Terruzzi, Anna Montesano, Pamela Senesi, Isabella Villa, Anita Ferraretto, Michela Bottani, Fernanda Vacante, Alice Spinello, Simona Bolamperti, Livio Luzi, Alessandro Rubinacci

**Affiliations:** ^1^Department of Biomedical Sciences for Health, Università degli Studi di Milano, Via Luigi Mangiagalli, 31, 20133 Milano, Italy; ^2^Metabolism Research Center, IRCCS Policlinico San Donato, Piazza Edmondo Malan, 2, 20097 San Donato Milanese, Italy; ^3^Bone Metabolism Unit, San Raffaele Scientific Institute, Via Olgettina 60, 20132 Milano, Italy; ^4^IRCCS Istituto Ortopedico Galeazzi, Laboratory of Experimental Biochemistry & Molecular Biology, Via Riccardo Galeazzi 4, 20161 Milano, Italy

## Abstract

Bone fragility and associated fracture risk are major problems in aging. Oxidative stress and mitochondrial dysfunction play a key role in the development of bone fragility. Mitochondrial dysfunction is closely associated with excessive production of reactive oxygen species (ROS). L-Carnitine (L-C), a fundamental cofactor in lipid metabolism, has an important antioxidant property. Several studies have shown how L-C enhances osteoblastic proliferation and activity. In the current study, we investigated the potential effects of L-C on mitochondrial activity, ROS production, and gene expression involved in osteoblastic differentiation using osteoblast-like cells (hOBs) derived from elderly patients. The effect of 5mM L-C treatment on mitochondrial activity and L-C antioxidant activity was studied by ROS production evaluation and cell-based antioxidant activity assay. The possible effects of L-C on hOBs differentiation were assessed by analyzing gene and protein expression by Real Time PCR and western blotting, respectively. L-C enhanced mitochondrial activity and improved antioxidant defense of hOBs. Furthermore, L-C increased the phosphorylation of Ca^2+^/calmodulin-dependent protein kinase II. Additionally, L-C induced the phosphorylation of ERK1/2 and AKT and the main kinases involved in osteoblastic differentiation and upregulated the expression of osteogenic related genes, RUNX2, osterix (OSX), bone sialoprotein (BSP), and osteopontin (OPN) as well as OPN protein synthesis, suggesting that L-C exerts a positive modulation of key osteogenic factors. In conclusion, L-C supplementation could represent a possible adjuvant in the treatment of bone fragility, counteracting oxidative phenomena and promoting bone quality maintenance.

## 1. Introduction

Bone participates in mineral homeostasis and fulfills its biomechanical functions through the process of bone remodeling. During aging, the remodeling process is no longer balanced and a decline in bone-forming cells compared to bone-resorbing cells activity occurs, leading to bone mass loss and quality deterioration. Several pathogenic mechanisms contribute to these age-related bone features, but the decreased differentiation of mesenchymal stem cells into osteoblasts and/or the senescence of the mature osteoblasts is considered as major contributors [1–3].

Many studies recognize the key role of mitochondria activity in ensuring the efficiency of cellular metabolic functions such as adenosine triphosphate (ATP) production* via* oxidative phosphorylation and electron transport chain (ETC), calcium homeostasis, reactive oxygen species (ROS) generation, and cellular apoptosis regulation [4, 5]. Since bone remodeling, in particular osteoblast differentiation, requires great amount of energy, efficient mitochondria are vital for bone formation and bone mass maintenance. In fact, during osteoblast differentiation, strong mitochondrial biogenesis was observed, accompanied by increased ATP production as well as decreased mitochondrial stress [6].

Mitochondrial key role in aging is linked to their essential contribution in the production and control of ROS levels. At low concentrations, ROS act as signal regulating numerous cellular functions: nuclear transcription activity, cell redox maintenance, cell growth, and differentiation [7, 8]. Nevertheless, the excessive increase of ROS can cause DNA damage and mitochondrial dysfunction contributing to the development of various pathological conditions [9]. In fact, oxidative stress has been associated with osteoblast damage and bone diseases in aging [6, 10].

Recent interest has been developed on nutraceuticals for their possibility to modulate osteoblast activity and oxidative phenomena. L-carnitine (L-C), a cofactor in the *β*-oxidation of fatty acids and a shuttle for the acetyl groups through the mitochondrial membrane [11], has been shown to stimulate human osteoblast functions [12] and intracellular calcium signaling [13]. In particular, L-C, increasing the performance of mitochondria [14], might provide positive outcome in high-energy demanding organs, such as muscle and bone. Noteworthy, L-C displays a direct effect on human osteoblast by significantly increasing osteoblast activity and proliferation, as well as the expression of collagen type I, bone sialoproteins (BSPs), and osteopontin (OPN) [12, 14].

Lately, L-C was shown to have antioxidant activity counteracting age-associated mitochondrial dysfunction in rats [15, 16] and to keep the balance of mitochondrial production of ROS in different type of cells [17, 18]. Recently, we demonstrated L-C ability to prevent oxidative stress, to counteract mitochondrial dysfunction and to accelerate the differentiation of C2C12 myoblasts by inducing myotubes formation and hypertrophy [19].

We performed the following study with the aim to characterize the effect of L-C in human trabecular bone derived osteoblasts (hOB). We evaluated L-C potential in modulating the balance between mitochondrial-mediated ROS production and catabolism. We investigated also L-C potential effect in modulating the downstream signaling of Ca^2+^ and in stimulating the expression of osteogenic genes. Given the interest on nutraceuticals as possible adjuvants in the current therapies for osteoporosis, this study could help in characterizing the molecular mechanisms underlying L-C positive effects on bone cells, thus supporting L-C beneficial effects in the treatment of bone fragility in the elderly.

## 2. Materials & Methods

### 2.1. Materials

All reagents and media were purchased from Sigma Chemical Co. (St. Louis-MO, USA). Primary antibodies against Calnexin (H-70), GAPDH (FL-335), AKT (C-20), CaMKII (M-176), pCaMKII*α* (Thr286), ERK1 (K-23), ERK2 (C-14), pERK1/2 (E-4), OPN (K-20), and SOD2 (FL-222) were purchased from Santa Cruz Biotechnology (Heidelberg, Germany). Primary antibody against Phospho-AKT (Ser473-D9E-XP™) was purchased from Cell Signaling Technology (Danvers-MA, USA). Peroxidase-conjugated secondary antibodies for Western blot analysis and FITC- or Rhodamine-conjugated antibodies for immunofluorescence study were purchased from Santa Cruz Biotechnology (Heidelberg, Germany). Fluorescently-labeled Phalloidin (AlexaFluor®488-Invitrogen) was purchased from Life Technologies (Carlsbad, CA, USA). CytoPainter Mitochondrial Staining Kit–Green (AB 112143) was purchased from Prodotti Gianni (Milano, Italy), and Cell ROX® Oxidative Stress Reagents Kit (C10443) was purchased from Thermo Fisher Scientific, Life Technologies Italia (Monza, Italy).

### 2.2. Human Osteoblast-Like Cells (hOBs) Cultures

According to a modified version of the Gehron-Robey & Termine procedure [20], human osteoblast cultures were obtained from waste material of female patients during orthopedic surgery for degenerative diseases or traumatic fractures of the femoral neck requiring osteotomy. The protocol was approved by the Institutional Ethical Committee (Protocol BMU-WNT, 25.03.2008) and the patients (aged 71–82yr) signed the informed consent for the use of the waste material. None of them was affected by any malignant bone diseases. The effects studied were not affected by the age of the donors. Briefly, the trabecular bone was cut into small pieces, rinsed, and incubated with rotation at 37°C for 30 min with 0.5 mg/ml type IV collagenase. The bone pieces were then placed in 25cm^2^ flasks and cultured in Iscove's modified medium (IMDM) containing 10% FBS, 100 U/ml penicillin, 100 *μ*g/ml streptomycin, 50 U/ml mycostatin, and 0.25*μ*g/ml amphotericin B until confluence. Cells were used at first passage to reduce the possibility of phenotype changes.

### 2.3. Reverse Transcription and Semiquantitative Real TimePCR

After 24h of serum starvation, hObs were treated for different times (1, 3, and 6h) with L-C (5mM). Total RNA from confluent hOBs was extracted using TRIzol reagent (Thermo Fisher Scientific Inc., Waltham, MA USA), according to the manufacturer's instructions. One *μ*g of total RNA was reverse transcribed to cDNA using oligodT primers and M-MLV reverse transcriptase (Promega Corporation, Madison, WI, USA). mRNAs expression of osteogenic genes RUNX2, osterix (OSX), bone-sialo protein (BSP), osteopontin (OPN), and osteocalcin (BGP) was evaluated by Real Time PCR using primer-probe sets validated and purchased as “Assay-on-Demand” from Applied Biosystems (Thermo Fisher Scientific Inc., Waltham, MA USA) in singleplex PCR mix. Real time PCR reaction was performed in an ABI PRISM® 7900 Sequence Detection System (Thermo Fisher Scientific Inc., Waltham, MA USA). Gene expression was calculated with the 2^−ΔΔCt^ method and *β*-actin was used as housekeeping gene. Each experimental point was analyzed in three replicates and experiments were performed several times with cells obtained from different donors.

### 2.4. Western Blot Analysis

After 24 h of serum free culture, confluent hObs were stimulated with L-C (5 mM) for 5, 15, and 60 min and for 6h and 24h. Afterwards, hOBs cells were lysed with RIPA buffer supplemented with proteases and phosphatases inhibitors. Equal amounts (35 *μ*g) of protein, quantified using Thermo Scientific Pierce BCA Protein Assay Kit (Thermo Fisher Scientific, Milan, Italy), were separated on SDS-PAGE and transferred to nitrocellulose membrane (Amersham™ Protran®, Sigma Chemical Co., St. Louis-MO, USA). Immunoblots were performed as described previously [21]. Immunoreactive bands were detected by ECL system according to the manufacturer's protocol (Amersham Pharmacia Biotech, Piscataway, NJ, USA). Calnexin or GAPDH bands were used as loading controls. Scion Image software (Scion Corp., Frederick, MD, USA) was used to analyze and quantify bands on X-ray films. The results were then converted to fold change (FC) of the control.

### 2.5. Immunofluorescence Analysis

After having starved hObs for 24h, cells were treated for 3, 6, and 24 h with L-C (5 mM). hObs, fixed and permeabilized, were incubated in PBS with 1% BSA to avoid nonspecific binding of the antibody. Cells were then incubated with specific antibodies FITC or rhodamine conjugated, and nuclei were revealed with DAPI staining. The MITO CytoPainter mitochondrial indicator is a hydrophobic compound that easily permeates intact live cells and becomes trapped in mitochondria. Cell ROX® Oxidative Stress Reagents are fluorogenic probes designed to measure reactive oxygen species (ROS) in living cells that exhibit a strong fluorogenic signal during oxidation. Cells were analyzed using Nikon Eclipse 50I microscopy with Nis-Elements D 4.00 software (Nikon Instruments Europe BV, Netherlands). Quantification of immunofluorescence signal was performed by using Image J program (http://imagej.nih.gov/ij/).

### 2.6. Cell-Based Antioxidant Activity (CAA) Assay

The antioxidant activity of L-C in hOBs was evaluated with the cell-based antioxidant activity (CAA) assay [22]. This cell-based assay uses the fluorescent probe 2',7'-dichlorofluorescin diacetate (DCFH-DA) to determine the radical scavenging activity of a compound. DCFH-DA 60 *μ*M in HBSS solution (containing 10 mM Hepes) was added for 20 min to fully confluent cells in a 96 wells plate. At the end of incubation, 5 mM L-C or 5 mM L-C + 500 *μ*M 2,2'-azobis(2-methylpropionamide) dihydro-chloride (AAPH), an oxidant able to generate peroxyl radicals, was added to cells and the fluorescence emission at 538 nm (excitation at 485 nm) was continuously recorded for 120 min (10 min time points) by a plate reader (Wallac Victor2 1420 Multilabel Counter plate reader (Perkin Elmer, Beaconsfield, UK). Further samples in the cell plate were control (CRT), CRT + AAPH, CRT + L-glutathione (GLUT), and GLUT + AAPH samples. CRT represented cells incubated only with DCFH-DA; CRT + AAPH cells incubated with DCFH-DA and 500 *μ*M AAPH; CRT + GLUT cells treated with DCFH-DA and 750 *μ*M GLUT; GLUT + AAPH cells treated with DCFH-DA, 750 *μ*M GLUT, and 500 *μ*M AAPH.

The CAA value was calculated for each condition as follows:

CAA  unit = 100 − (∫SA  ∕∫CA) × 100

where ∫SA is the integrated area under the sample fluorescence versus time curve and ∫CA is the integrated area of the CRT curve, after subtracting the background value from the fluorescence readings. Positive CAA values indicate antioxidant activity vs CRT (CAA value of 0), while negative CAA values indicate oxidant activity vs CRT.

### 2.7. Statistical Analysis

Results are shown as the mean ± SD or ± SEM. Statistical analysis were performed with Prism v 7.00 (GraphPad Software, San Diego, CA, USA) and SPSS 20 (Chicago, IL, USA). Statistically significant differences were determined using either Student's* t* test or ANOVA tests (Kruskal–Wallis test, two-ways ANOVA) followed by appropriate multiple-comparison test: Dunn's post test, or Bonferroni post hoc t-test. Results were considered significant when P ≤ 0.05.

## 3. Results

### 3.1. hOBs Mitochondrial Response to L-Carnitine Stimuli

**Figure 1:**
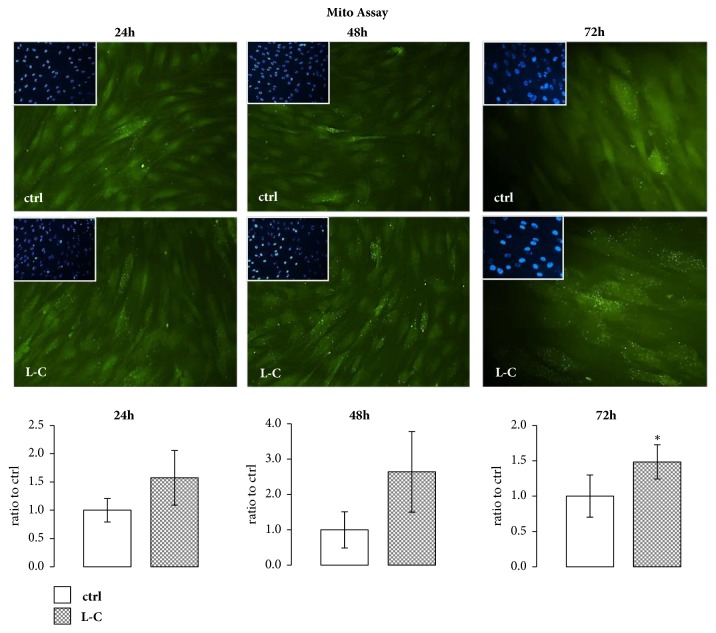
**hOBs mitochondrial response to L-Carnitine (L-C) treatment. **After 24, 48, and 72h of treatment, the CytoPainter Mitochondrial fluorescence signal associated with active mitochondria was increased in L-C-treated hOBs compared to controls (ctrl), reaching significance at 72h. Mito signal (green), nuclei (DAPI). The quantified signal was normalized for the total nuclei number (magnification: 20X for 24h and 48h; 40X for 72h). Data are the mean ± SD of six experiments performed with cells obtained from different donors. ANOVA for nonparametric data (Kruskal–Wallis) with Dunn's multiple comparison test: ^**∗**^p< 0.05 vs control (ctrl).

In order to evaluate L-C effects on mitochondrial activity, hOBs were treated with L-C (5mM) for 24h, 48h, and 72h. CytoPainter Mitochondrial Staining Assay showed that L-C increased the intensity of the signal associated to active mitochondria after 24, 48 and 72h of treatment compared to controls, reaching significance (p<0.05) after 72h (Figure 1). The capacity of L-C to modulate mitochondrial activity in hOBs suggests that L-C could support the antioxidant activity of osteoblasts.

### 3.2. L-Carnitine Decreases Oxidative Stress and Increases Antioxidative Defense in hOBs

**Figure 2:**
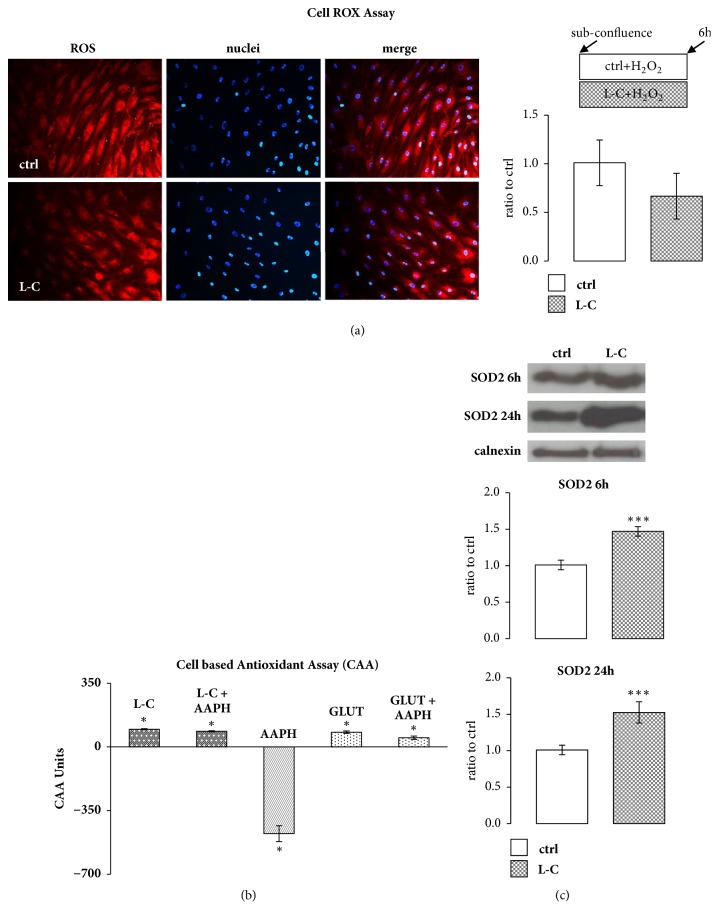
**L-Carnitine (L-C) decreases oxidative stress and increases anti oxidative defense in hOBs. **(a): hOBs treated with or without 5 mM L-C for 6h were incubated in oxidative condition (500 mM H_2_0_2_) with Cell ROX® Orange Reagent. L-C induced a decrease, although not significant, in ROS fluorescence signal. ROS signal (red), nuclei (DAPI), and merge (magnification: 20X). The quantified signal was normalized for the total nuclei number. (b): 5mM L-C treatment was able to enhance antioxidant hOBs capacity even in presence of the pro-oxidant agent AAPH. Glutathione was used as antioxidant activity control (CAA assay: Each bar reported the CAA values vs untreated cells, represented by the 0 line; see materials and methods; ^*∗*^p<0.01, two-way ANOVA with Bonferroni post-test). (c): Representative Western blot and relevant quantification of SOD2 protein: after 6 and 24h, L-C induced a significant rise of SOD2 protein content. Data are the mean ± SD of six experiments performed with cells obtained from different donors. For Western blot and Immunofluorescence studies: Student's t test: ^*∗∗∗*^p<0.001 vs control (ctrl).

To evaluate the response of hOBs to oxidative stress, the cells were exposed to 500 *μ*M H_2_0_2_ and L-C treatment for 6h. L-C induced a decrease in the mitochondrial production of ROS, compared to controls. Considering that the observed decrease in ROS production did not reach a statistical significance (Figure 2(a)), to evaluate the possible antioxidant activity of L-C in hOBs, we performed a cell based antioxidant assay (CAA). The results showed a statistically significant antioxidant effect of L-C compared to the controls (CAA value = 97 ± 3, p<0.01). Moreover, when the oxidant molecules 2,2'-azobis(2-methylpropionamide) dihydro-chloride (AAPH, 500 *μ*M) was added together with L-C, the antioxidant activity of L-C was still detectable, thus indicating its antagonistic effect towards the oxidative stress induced by AAPH (CAA value = 87 ± 3; p<0.01). Glutathione (GLUT), a well-characterized antioxidant compound, was used as a positive control. GLUT demonstrated a significant antioxidant activity both alone (CAA value of + 81 ± 7, P< 0.01) and in the presence of AAPH (CAA value of + 50 ± 10, p<0.01) (Figure 2(b)) comparable with the effect observed with L-C.

Furthermore, considering the pivotal role of superoxide dismutase 2 (SOD2) in limiting ROS production in oxidative stress conditions in mitochondria, we evaluated the effect of L-C on SOD2. L-C induced a significant increase in SOD2 protein content after 6h and 24h treatment compared to controls (p<0,001, Figure 2(c)).

### 3.3. L-Carnitine Action on Intracellular Calcium Pathway

**Figure 3:**
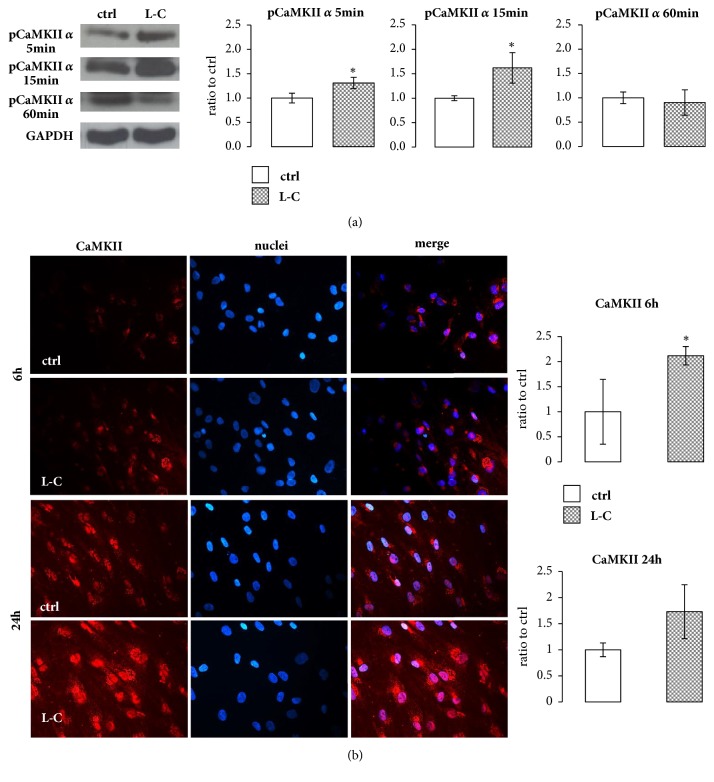
**L-Carnitine (L-C) effect on intracellular calcium pathway. **(a): Representative Western blot and relevant quantification of CaMKII*α* phosphorylation in hOBs treated with 5 mM L-C. CaMKII*α* activation was observed at 5 and 15 min. (b): Immunofluorescence assay and quantification of total CaMKII protein after 6 and 24h. L-C enhanced the protein content of total CaMKII compared to controls after 6h. CaMKII (red), nuclei (DAPI), and merge. The quantified signal was normalized for the total nuclei number (magnification: 40X). Data are the mean ± SD of six experiments performed with cells obtained from different donors. For immunofluorescence and Western blot analyses: Student's* t* test: ^*∗*^p<0.05 vs control (ctrl).

Considering that our group recently showed that L-C modulates calcium signaling in hOBs [13], we analyzed the effect of L-C on the downstream signaling of Ca^2+^, by investigating the activation of the calcium-dependent enzyme Ca^2+^/calmodulin-dependent protein kinase II (CaMKII). L-C was able to significantly increase the phosphorylation of CaMKII*α* after 5 min and 15 min of treatment (p<0.05). After 60 min the effect on CaMKII*α* phosphorylation returned to the levels of untreated cells (Figure 3(a)). Moreover, immunofluorescence staining showed that L-C significantly increased the protein content of total CaMKII after 6h (p<0.05) compared to controls. The increase was still present after 24h, but not statistically significant, probably due to the variability of the data (Figure 3(b)).

### 3.4. L-Carnitine Effect on Intracellular Signaling in hOBs

**Figure 4:**
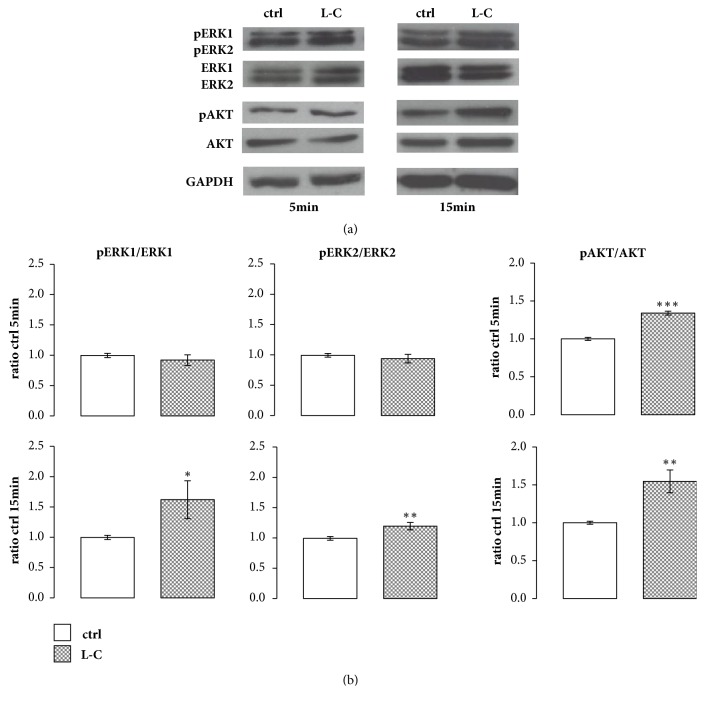
**L-Carnitine (L-C) effect on intracellular signaling in hOBs. **(a): Representative Western blot of ERKs and AKT activation in hOBs after 5 and 15 min of treatment with 5 mM L-C. (b): Relevant quantification of ERK1/2 and AKT phosphorylation. L-C induced a significant phosphorylation of ERK1/2 at 15 min and of AKT at 5 and 15 min. Data are the mean ± SD of six experiments performed with cells obtained from different donors. Student's t test: ^*∗*^p<0.05, ^*∗∗*^p<0.01,and ^*∗∗∗*^p<0.001 vs control (ctrl).

As Ca^2+^ and calmodulin induce the activation of MAPK signalling in mammalian cells [23], we investigated the activation of both ERK and AKT after L-C treatment in hOBs. L-C induced a significant increase in the phosphorylation of both ERK1 (p<0.05) and ERK2 (p<0.01) after 15 min of treatment compared to controls, as shown by the ratios of pERK1/ERK1 and pERK2/ERK2 reported in Figure 4(a). Similarly, L-C significantly increased the phosphorylation of AKT after 5 min (p<0.001) and 15 min (p<0.01) of treatment compared to controls (Figure 4(b)).

Considering that ERK and AKT have a pivotal role in the osteogenic lineage differentiation, their activation suggests a possible involvement of L-C in hOBs differentiation and leads us to investigate the possible modulation of key osteogenic factors after L-C treatment.

### 3.5. L-Carnitine Effect on the Expression of Osteogenic Related Genes

**Figure 5:**
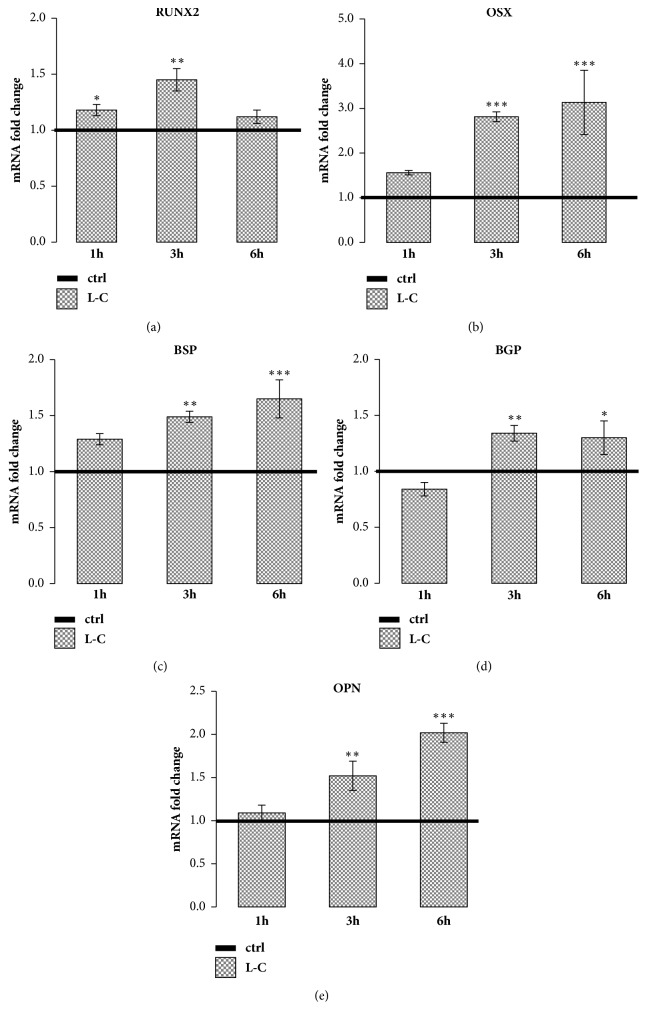
**L-Carnitine (L-C) increased the expression of osteogenic-related genes and of matrix proteins genes. **L-C (5mM) stimulated significantly the gene expression of RUNX2 (a) at 1 and 3h and of OSX (b), BSP (c), BGP (d) and OPN (e) at 3 and 6h compared to control (straight line). Data are the mean ± SEM of six to nine experiments performed with cells obtained from different donors. ANOVA for nonparametric data (Kruskal–Wallis) with Dunn's multiple comparison test: ^*∗*^p<0.05, ^*∗∗*^p<0.01, and ^*∗∗∗*^p<0.001 vs control.

**Figure 6:**
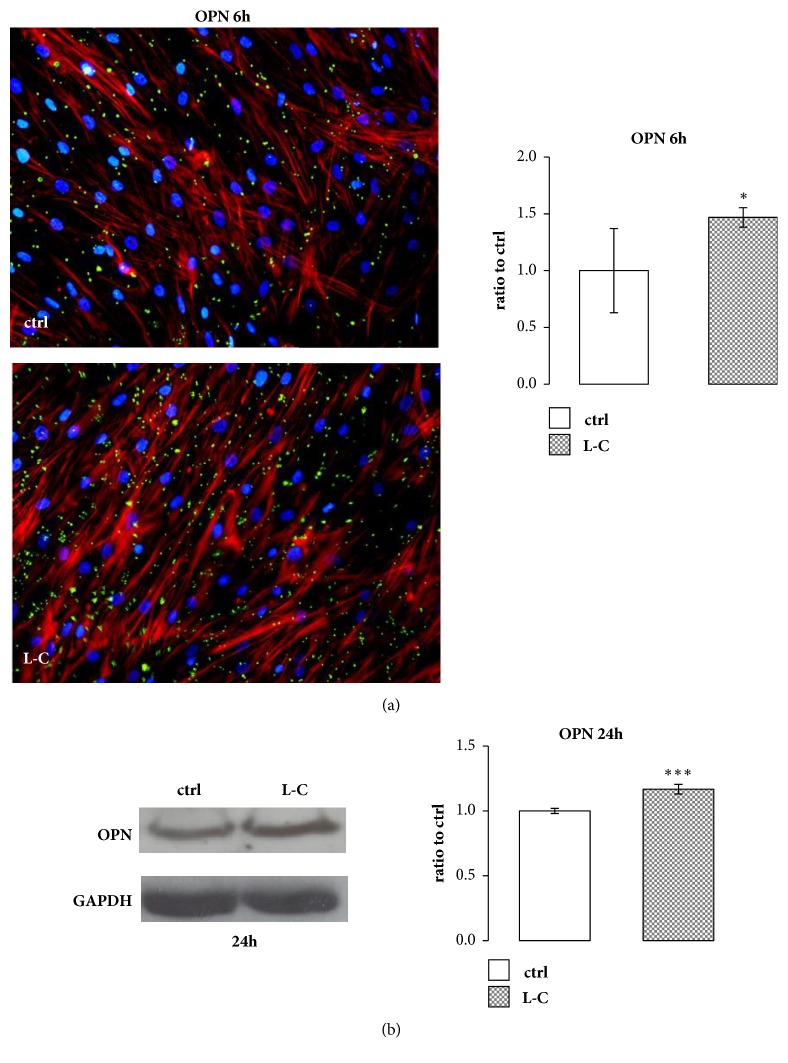
**L-Carnitine (L-C) effect on OPN protein. **(a): Immunofluorescence staining of OPN protein after 6h of 5 mM L-C treatment and relevant quantification showed an increase in OPN protein level compared to control (ctrl). Osteoblastic morphology was analyzed by Phalloidin (red) and OPN (green) staining. The quantified signal was normalized for the total nuclei number (magnification: 20X). (b): Representative Western blot and relevant quantification of OPN protein content increased after 24h of 5 mM L-C stimuli. Data are the mean ± SD of six to nine experiments performed with cells obtained from different donors. Student's t test: ^*∗*^p<0.05, ^*∗∗∗*^p<0.001 vs control (ctrl).

L-C treatment stimulated mRNA expression of RUNX2 and OSX, two main transcription factors that modulate the synthesis of osteoblast specific proteins. L-C induced a significant increase of RUNX2 at 1h (p<0.05) and 3h (p<0.01) and of OSX at 3h and 6h of treatment (p<0.001, Figures 5(a) and 5(b)). Three and 6h L-C treatments were also able to significantly stimulate BSP (p<0.01, p<0.001), BGP (p<0.01, p<0.05), and OPN (p<0.01, p<0.001) mRNA expression compared to controls (Figures 5(c), 5(d), and 5(e)). Immunofluorescence staining and Western blot showed that OPN protein was increased after 6h (p<0.05) and 24h (p<0.001) after L-C treatment as well (Figure 6).

### 3.6. ERK Involvement in the Increased Expression of Osteogenic Related Genes after L-Carnitine Treatment

**Figure 7:**
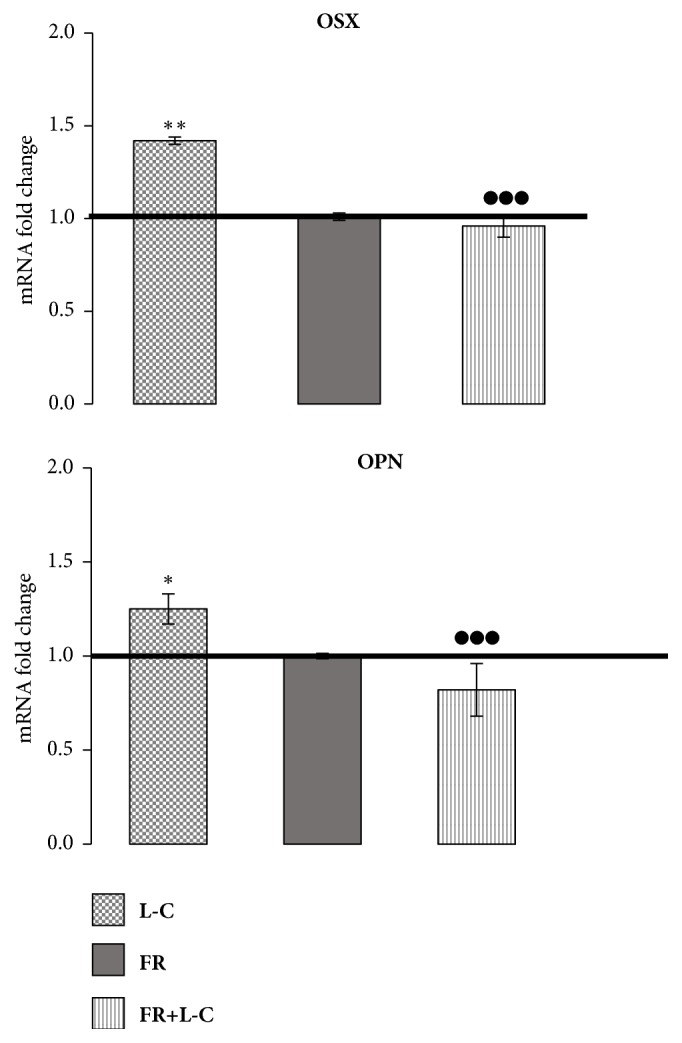
**Effect of the inhibition of ERKs signaling on L-Carnitine (L-C) induced gene expression. **The increase in OSX and OPN mRNA expression 3h after L-C was prevented by pretreating hOBs 30 min before L-C with 10*µ*M FR180204, an inhibitor of ERKs activity. ANOVA for nonparametric data (Kruskal–Wallis) with Dunn's multiple comparison test: ^*∗*^p<0.05, ^*∗∗*^p<0.01 vs control (ctrl); ^●●●^p<0.001 vs L-C.

In order to investigate the role of ERK signaling in the observed increase in osteogenic genes expression after L-C treatment, we used FR180204, a selective inhibitor of the kinase activity of ERK1 and ERK2 [24]. Pretreatment with 10*μ*M FR180204 30 min before 5mM L-C prevented the increase in RUNX2 and OPN expression by L-C (Figure 7), suggesting that ERKs signaling is involved in L-C induced increase in the expression of the osteogenic genes.

## 4. Discussion

In this study, we showed that L-C enhances the overall performance of the mitochondria and protects against oxidative stress. Moreover, L-C activates CaMKII and ERKs/AKT signaling cascade thus favouring the expression of osteogenic genes.

Mitochondria have major roles in biological processes ranging from cellular oxygen sensing to the regulation of calcium levels [25]. The age related structural deterioration of bone, due to a dysregulation of bone remodelling, could be ascribed, at least in part, to reduced energy supply for altered mitochondria biogenesis and activity [6]. Our study, showing that L-C enhances the overall performance of the mitochondria, suggests that L-C could support the fulfillment of the high metabolic demand of osteoblasts during bone formation.

A defect in the control of mitochondrial ROS concentration generates an imbalance between the production and the degradation of superoxide radicals [26], which exceeds the osteoblasts detoxification capacity, and inhibits the expression of osteogenic genes and matrix mineralization [27]. The L-C capacity to increase the mitochondrial production of SOD2 and to decrease osteoblast ROS content, here shown, suggests that the mitochondrial-mediated balance between ROS production and catabolism is improved by L-C treatment. In fact, to prevent damages due to an accumulation of ROS, mitochondria dismutate unstable superoxide anions into the more stable products O_2_ and H_2_O_2_ through the activity of SOD2 activity. SOD2 is the main mitochondrial antioxidant defense and plays a pivotal role during osteoblast differentiation as well. SOD2 deficiency generates an osteoblast dysfunction, whereas its overexpression enhances the differentiation of primary osteoblasts derived from SIRT3-deficient mice, which are characterized by impaired mitochondrial homeostasis and increased mitochondrial ROS [6]. Considering that reducing the intracellular ROS levels is the primary defense against the cell injury induced by oxidative stress, we can speculate that L-C could potentially counteract the excessive ROS accumulation that favors the altered osteoblast metabolism during aging. In addition, SOD2 capacity to reduce ROS levels is able to inhibit the osteoclasts differentiation process, in which ROS play a role as second messenger [28].

In the present study, we also showed that L-C activates CaMKII and ERKs/AKT signaling cascade to induce osteogenic gene expression. CaMKII is an ubiquitous protein. The CaMKII *α* isoform is mainly expressed in osteoblasts and has a role in their differentiation [29]. In mammalian cells, CaMKII signaling induces the activation of MAP kinases [23], such as ERKs and AKT. These two pathways are essential for osteoblast growth and differentiation [30] and are involved in the modulation of integrin levels at cell surface, thus contributing to cell adhesion [31]. Moreover, ERKs increase the expression of RUNX2 [32], the main transcription factor of osteoblasts differentiation, thus regulating the deposition of bone matrix proteins [33]. Our results showing that L-C upregulates both RUNX2 and OSX gene expressions,* via* ERK pathway, support a role of L-C in favoring osteogenic cell functions. Noteworthy, L-C also activates AKT, a serine/threonine kinase present in all cells that, when activated, mediates downstream responses, including cell survival, growth, and proliferation by phosphorylating a range of intracellular proteins. When activated in osteoblast, AKT enhances osteoblast differentiation by promoting RUNX2 protein stability [34]. It is therefore likely that AKT activation by L-C might contribute to the observed positive effect of the compound on osteogenesis.

Finally, L-C stimulates the expression of noncollagenous proteins such as BSP, BGP, and OPN. In particular, BSP is involved in the regulation of hydroxyapatite crystal formation. BSP^−/−^ mice display impaired mineralization and endochondral bone development [35]. BGP is specifically produced by osteoblasts and, besides its endocrine role [36], it is a marker of mature osteoblast. OPN influences the mechanical properties of bone. OPN^−/−^ mice are characterized by reduced bone strength, although their overall bone phenotype is normal [37]. Given the critical role of these noncollagenous proteins in determining bone mineralization, elasticity, and strength [38], we can speculate that their induction in hOBs by L-C might have a beneficial effect on the osteoporotic bone. In osteoporotic condition, noncollagenous proteins are indeed modified and their amount is reduced with aging, thus affecting mechanical competence [39]. This hypothesis is further supported by the evidence that L-C and its derivatives, by accelerating the recovery of normal BV/TV, have a beneficial effect on trabecular bone in a hypocalcemic diet-induced mouse model of osteoporosis. [40].

## 5. Conclusions

**Figure 8:**
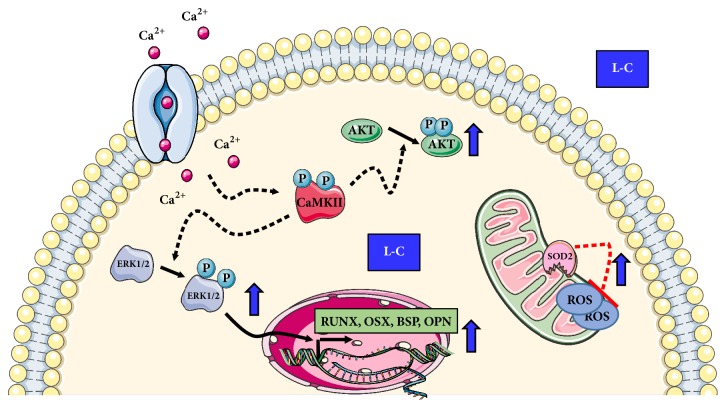
Schematic summary of L-Carnitine (L-C) activity in human osteoblasts.

In conclusion, we showed that, in hOBs, L-C improves mitochondrial antioxidant activity and stimulates several signaling pathways involved in osteogenesis. Our study, by further characterizing L-C effects in osteoblasts (summarized in Figure 8), suggest a role for L-C as a potential candidate in counteracting the decline of bone mass in skeletal senescence.

## Data Availability

The experimental data used to support the findings of this study are included within the article.

## Abbreviations

BSP: Bone sialoprotein
L-C: L-Carnitine
CaMKII: Ca^2+^/calmodulin-dependent protein kinase II
CAA: Cell-based antioxidant activity assay
DCFH-DA: Fluorescent probe 2',7'-dichlorofluorescin diacetate
ERK1/2: Extracellular signal–regulated kinase 1/2
FC: Fold change
FBS: Fetal Bovine Serum
GAPDH: Glyceraldehyde-3-Phosphate Dehydrogenase
hObs: Human osteoblasts
OPN: Osteopontin
OSX: Osterix
pCaMKII *α*: Ca^2+^/calmodulin-dependent protein kinase II isoform *α*
ROS: Reactive oxygen species
RUNX2: Runt-related transcription factor 2
SOD2: Superoxide dismutase 2.

## References

[1] Manolagas S. C., Parfitt A. M. (2010). What old means to bone. *Trends in Endocrinology & Metabolism*.

[2] Kassem M., Marie P. J. (2011). Senescence-associated intrinsic mechanisms of osteoblast dysfunctions. *Aging Cell*.

[3] Roholl P. J. M., Blauw E., Zurcher C., Dormans J. A. M. A., Theuns H. M. (1994). Evidence for a diminished maturation of preosteoblasts into osteoblasts during aging in rats: An ultrastructural analysis. *Journal of Bone and Mineral Research*.

[4] Tait S. W., Green D. R. (2010). Mitochondria and cell death: Outer membrane permeabilization and beyond. *Nature Reviews Molecular Cell Biology*.

[5] Nicholls D. G. (2005). Mitochondria and calcium signaling. *Cell Calcium*.

[6] Gao J., Feng Z., Wang X. (2017). SIRT3/SOD2 maintains osteoblast differentiation and bone formation by regulating mitochondrial stress. *Cell Death & Differentiation*.

[7] Ristow M., Schmeisser K. (2014). Mitohormesis: Promoting health and lifespan by increased levels of reactive oxygen species (ROS). *Dose-Response*.

[8] Finkel T. (2011). Signal transduction by reactive oxygen species. *The Journal of Cell Biology*.

[9] Brieger K., Schiavone S., Miller F. J., Krause K.-H. (2012). Reactive oxygen species: From health to disease. *Swiss Medical Weekly*.

[10] Birch-Machin M. A., Bowman A. (2016). Oxidative stress and ageing. *British Journal of Dermatology*.

[11] Pekala J., Patkowska-Sokoła B., Bodkowski R. (2011). L-carnitine—metabolic functions and meaning in humans life. *Current Drug Metabolism*.

[12] Colucci S., Mori G., Vaira S. (2005). L-carnitine and isovaleryl L-carnitine fumarate positively affect human osteoblast proliferation and differentiation in vitro. *Calcified Tissue International*.

[13] Ferraretto A., Bottani M., Villa I. (2018). L-Carnitine activates calcium signaling in human osteoblasts. *Journal of Functional Foods*.

[14] Marcovina S. M., Sirtori C., Peracino A. (2013). Translating the basic knowledge of mitochondrial functions to metabolic therapy: Role of L-carnitine. *Translational Research*.

[15] Kumaran S., Subathra M., Balu M., Panneerselvam C. (2005). Supplementation of L-carnitine improves mitochondrial enzymes in heart and skeletal muscle of aged rats. *Experimental Aging Research*.

[16] Bernard A., Rigault C., Mazue F., Le Borgne F., Demarquoy J. (2008). L-carnitine supplementation and physical exercise restore age-associated decline in some mitochondrial functions in the rat. *The Journals of Gerontology. Series A, Biological Sciences and Medical Sciences*.

[17] Le Borgne F., Ravaut G., Bernard A., Demarquoy J. (2017). L-carnitine protects C2C12 cells against mitochondrial superoxide overproduction and cell death. *World Journal of Biological Chemistry*.

[18] Benedini S., Perseghin G., Terruzzi I. (2009). Effect of L-acetylcarnitine on body composition in HIV-related lipodystrophy. *Hormone and Metabolic Research*.

[19] Montesano A., Senesi P., Luzi L., Benedini S., Terruzzi I. (2015). Potential therapeutic role of L-carnitine in skeletal muscle oxidative stress and atrophy conditions. *Oxidative Medicine and Cellular Longevity*.

[20] Robey P. G., Termine J. D. (1985). Human bone cells in vitro. *Calcified Tissue International*.

[21] Villa I., Senesi P., Montesano A. (2017). Betaine promotes cell differentiation of human osteoblasts in primary culture. *Journal of Translational Medicine*.

[22] Wan H., Liu D., Yu X., Sun H., Li Y. (2015). A Caco-2 cell-based quantitative antioxidant activity assay for antioxidants. *Food Chemistry*.

[23] Woods A., Dickerson K., Heath R. (2005). Ca^2+^/calmodulin-dependent protein kinase kinase-beta acts upstream of AMP-activated protein kinase in mammalian cells. *Cell Metabolism*.

[24] Ohori M., Kinoshita T., Okubo M. (2005). Identification of a selective ERK inhibitor and structural determination of the inhibitor-ERK2 complex. *Biochemical and Biophysical Research Communications*.

[25] Brookes P. S., Yoon Y., Robotham J. L., Anders M. W., Sheu S.-S. (2004). Calcium, ATP, and ROS: A mitochondrial love-hate triangle. *American Journal of Physiology-Cell Physiology*.

[26] Munro D., Treberg J. R. (2017). A radical shift in perspective: Mitochondria as regulators of reactive oxygen species. *Journal of Experimental Biology*.

[27] Arai M., Shibata Y., Pugdee K., Abiko Y., Ogata Y. (2007). Effects of reactive oxygen species (ROS) on antioxidant system and osteoblastic differentiation in MC3T3-E1 cells. *IUBMB Life*.

[28] Atashi F., Modarressi A., Pepper M. S. (2015). The role of reactive oxygen species in mesenchymal stem cell adipogenic and osteogenic differentiation: A review. *Stem Cells and Development*.

[29] Zayzafoon M., Fulzele K., McDonald J. M. (2005). Calmodulin and calmodulin-dependent kinase II*α* regulate osteoblast differentiation by controlling c-fos expression. *The Journal of Biological Chemistry*.

[30] Ge C., Cawthorn W. P., Li Y., Zhao G., Macdougald O. A., Franceschi R. T. (2016). Reciprocal control of osteogenic and adipogenic differentiation by ERK/MAP kinase phosphorylation of Runx2 and PPAR*γ* transcription factors. *Journal of Cellular Physiology*.

[31] Lai C.-F., Chaudhary L., Fausto A. (2001). Erk Is Essential for Growth, Differentiation, Integrin Expression, and Cell Function in Human Osteoblastic Cells. *The Journal of Biological Chemistry*.

[32] Fujita T., Azuma Y., Fukuyama R. (2004). Runx2 induces osteoblast and chondrocyte differentiation and enhances their migration by coupling with PI3K-Akt signaling. *The Journal of Cell Biology*.

[33] Vimalraj S., Arumugam B., Miranda P., Selvamurugan N. (2015). Runx2: Structure, function, and phosphorylation in osteoblast differentiation. *International Journal of Biological Macromolecules*.

[34] Choi Y. H., Kim Y.-J., Jeong H. M., Jin Y.-H., Yeo C.-Y., Lee K. Y. (2014). Akt enhances Runx2 protein stability by regulating Smurf2 function during osteoblast differentiation. *FEBS Journal*.

[35] Holm E., Aubin J. E., Hunter G. K., Beier F., Goldberg H. A. (2015). Loss of bone sialoprotein leads to impaired endochondral bone development and mineralization. *Bone*.

[36] Wei J., Karsenty G. (2015). An overview of the metabolic functions of osteocalcin. *Reviews in Endocrine and Metabolic Disorders*.

[37] Bailey S., Karsenty G., Gundberg C., Vashishth D. (2017). Osteocalcin and osteopontin influence bone morphology and mechanical properties. *Annals of the New York Academy of Sciences*.

[38] Alliston T. (2014). Biological regulation of bone quality. *Current Osteoporosis Reports*.

[39] Morgan S., Poundarik A. A., Vashishth D. (2015). Do non-collagenous proteins affect skeletal mechanical properties?. *Calcified Tissue International*.

[40] Patano N., Mancini L., Settanni M. P. (2008). L-carnitine fumarate and isovaleryl-L-carnitine fumarate accelerate the recovery of bone volume/total volume ratio after experimetally induced osteoporosis in pregnant mice. *Calcified Tissue International*.

